# Age and gender related differences in load-strain response in C57Bl/6 mice

**DOI:** 10.18632/aging.202350

**Published:** 2020-12-17

**Authors:** Hammad Mumtaz, Nuria Lara-Castillo, JoAnna M. Scott, Mark Begonia, Mark Dallas, Mark L. Johnson, Thiagarajan Ganesh

**Affiliations:** 1 University of Missouri-Kansas City, Department of Civil and Mechanical Engineering, Kansas, MO 64110, USA; 2 University of Missouri-Kansas City, School of Dentistry, Department of Oral and Craniofacial Sciences, Kansas, MO 64108, USA; 3 University of Missouri-Kansas City, Office of Research and Graduate Programs, Kansas, MO 64108, USA; 4 Virginia Polytechnic Institute and State University, Biomedical Engineering and Mechanics, Blacksburg, VA 24061, USA

**Keywords:** C57Bl/6, aging, digital image correlation, gender, mechanical loading

## Abstract

We examined the changes in mechanical strain response of male and female mouse tibia and ulna, using axial compression tests, to assess age-related changes in tibiae and ulnae by a non-contact strain measurement technique called the digital image correlation (DIC) and the standard strain gage. A unique aspect of the study was to compare bones from the same animal to study variations in behavior with aging. This study was conducted using male and female C57Bl/6 mice at 6, 12 and 22 months of age (N=6-7 per age and sex) using three load levels. The DIC technique was able to detect a greater number of statistically significant differences in comparison to the strain gaging method. Male ulna showed significantly higher DIC strains compared to strains captured from strain gage at all three levels of load at 6 months and in the lowest load at 12 months. DIC measurements revealed that the ulna becomes stiffer with aging for both males and females, which resulted in 0.4 to 0.8 times reduced strains in the 22-month group compared to the 6 month group. Male tibia showed three-fold increased strains in the 22 months group at 11.5 N load compared to 6 months group (p<.05).

## INTRODUCTION

Bone becomes more fragile and prone to fracture as we age [1–3]. Osteoporosis is a systemic skeletal disease resulting in low bone mass and micro architectural deterioration of bone tissue, which causes an increase in bone fragility and susceptibility to fracture. There are approximately 8.9 million fractures worldwide each year [4]. One of three women and one of five men over the age of 50 years has experienced an osteoporotic fracture [5, 6]. By 2050, the incidence of hip fracture alone is projected to increase by 240% in women and by 310% in men [7]. Due to this fact, the ability to maintain the quality of bone and to predict, diagnose and treat bone fragility has become an area of major focus in medical research.

Bone cells can detect and respond to mechanical loading. *In vivo* mechanical loading models are widely used to study the response of bone to load [8]. Bone responses to loading are related to the local strain stimulus [9] and hence understanding the relationship between the applied axial load (force) and the resulting strains that bone cells experience is important. Axial loading generates compression and bending in the mid-shaft region of long bones such as the tibia and ulna. Most tibial loading studies have been limited to young-adult (3–6 months old) mice. Studies in older mice are important to examine the potential for loading-based approaches to modulate age-related bone loss [10]. Although strain gage measurements are routinely used to characterize the strains at a single site on the tibia diaphysis, a thorough analysis has not been performed to assess possible differences in loading induced strain distributions with age [11]. In one study, Moustafa et al compared the mouse fibula to the tibia and ulna and demonstrated that the fibula is also a useful bone to assess the response of bone cells to mechanical loading [12]. In another study, Willie et al suggested that the trabecular bone loss in adults might not only be due to the reduced mechanoresponsiveness, but also that alterations in the load-induced strains within the bone may play a key role [13].

Strain gages applied to mouse ulna or tibia are commonly used to assess load strain relationships in *ex vivo* studies. However, based on our observations there are several limitations to this method such as the difficulty in the consistent application and positioning, limited analysis of a single bone region, and potential artificial stiffening of the mouse bone due to the application of adhesives and the strain gage to the bone surface. Digital Image Correlation (DIC) is an optical, non-contact method that accurately measures strains and circumvents many of the limitations of strain gaging. A speckle pattern is applied to the surface of the bone prior to loading while cameras track the position of the speckle pattern during loading. Videos from the loading session are broken down into a series of individual images, which are uploaded into specialized software that tracks the position of the speckles and then calculates the strains. DIC methods have been adopted in mouse femurs [14] and tibiae [15, 16]. In our previous study Begonia et al [17] used female TOPGAL mice (age 17.5 to 19.5 weeks) on a mixed C57Bl/6 X CD1 background and compared the strains in the radius and ulna, measured from the DIC and strain gage techniques. We concluded that DIC generally yielded a higher strain magnitude when compared to the conventional strain gaging. We suggested that the stiffening of the bone due to gluing the gages to the bone surface resulted in the underestimation of strains on the bone surfaces.

In this paper we have further examined the load strain relationship using C57Bl/6 mice ulna and tibia at 6, 12 and 22 months of age. We performed comparisons of DIC vs strain gage results based on age, gender, and bone type. These studies demonstrate age and gender differences in the load strain relationship, which varies depending on the type of bone and the method used (DIC or strain gaging). In addition, this paper has used data to study the behavior of different bones, from the same animal, with aging and gender, thereby providing valuable comparative data.

## RESULTS

**Figure 1:**
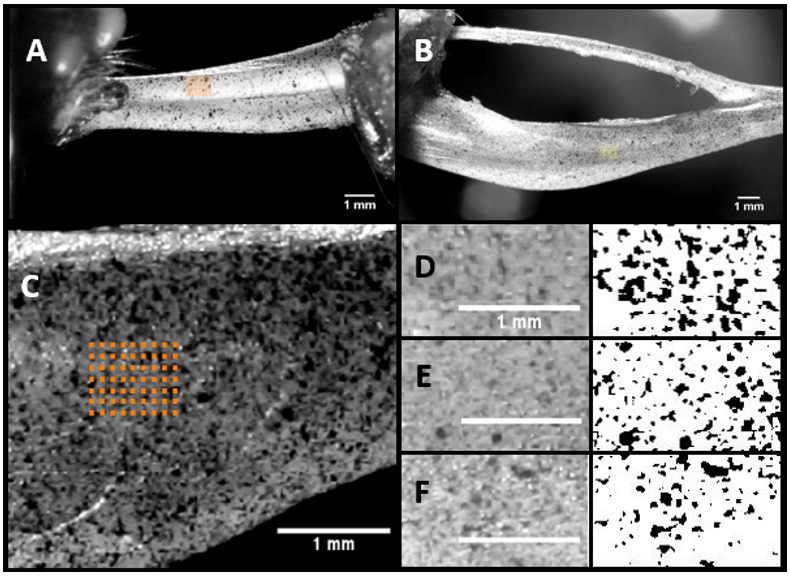
Representative image of the ROI selected in Ulna (**A**) and Tibia (**B**). ROI and a typical speckle pattern (**C**) and the regions selected (**D**), (**E**), and (**F**) for the computation of the speckle density using ImageJ.

The peak to peak strains were measured at specific regions of the bone which is termed as region of interest (ROI). The maximum strain value is the difference between the two peak values in a cyclic loading situation. Figure 1A and 1B shows a representative image with the actual ROI position selected for DIC strain measurements, which coincides with the position of the strain gage.

Figure 1C shows the ROI and its speckle in detail along with the ROI locations (Figure 1D–1F) selected for the estimation of speckle density from three different specimen. The images were converted into 8-bit images (right side) and the threshold was applied using ImageJ. The speckle density was calculated for all the three samples and the average was found to be 14.49%.

### Tibia – strain gauge vs. DIC

### Female tibia

**Figure 2:**
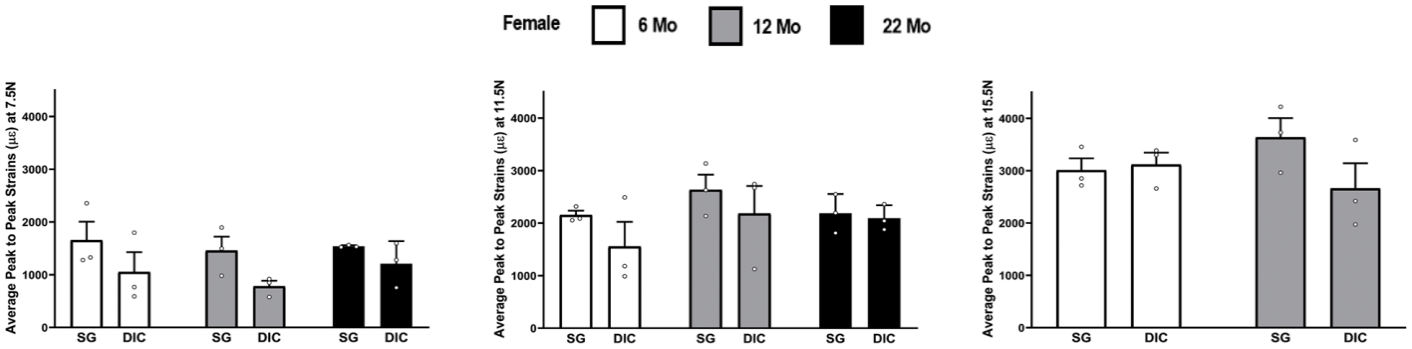
**Strains captured by Digital Image Correlation (DIC) and Strain Gage in female tibiae (data are mean ± standard error) at load levels of 7.5 N, 11.5 N and 15.5 N.**

Figure 2 shows the strain response in female tibia obtained from the DIC and strain gaging methods at three different levels of load, namely 7.5 N, 11.5 N and 15.5 N. The 22-month female tibiae were tested only up to a load level of 11.5 N due to their failure/fracture at higher loads. The strain gage measurements showed higher mean strain values than DIC measurements in almost all age and load levels. However, none of the differences were found to be statistically significant. The load strain relationship for female tibia is shown in Supplementary Figure 1.

### Male tibia

**Figure 3:**
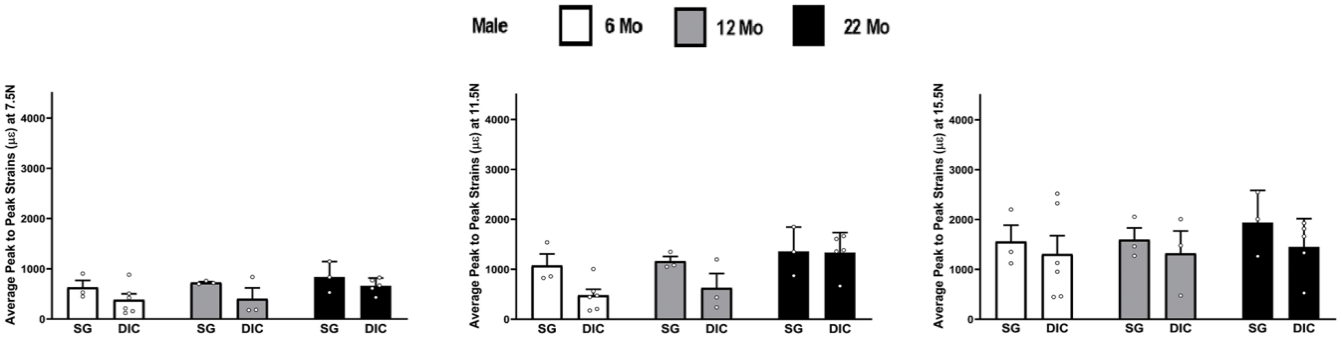
**Strains captured by Digital Image Correlation (DIC) and Strain Gage in male tibiae at load levels of 7.5 N, 11.5 N and 15.5 N.**

The strain values obtained from DIC and strain gaging in male tibiae at three different levels of load are shown in Figure 3. Like females, strains captured from the strain gage were higher compared to the DIC measurements. However, the average strain values showed no statistical differences at different ages between the strain gage and DIC methods. The load strain relationship for male tibia is shown in Supplementary Figure 2.

### Ulna – strain gauge vs. DIC

### Female ulna

**Figure 4:**
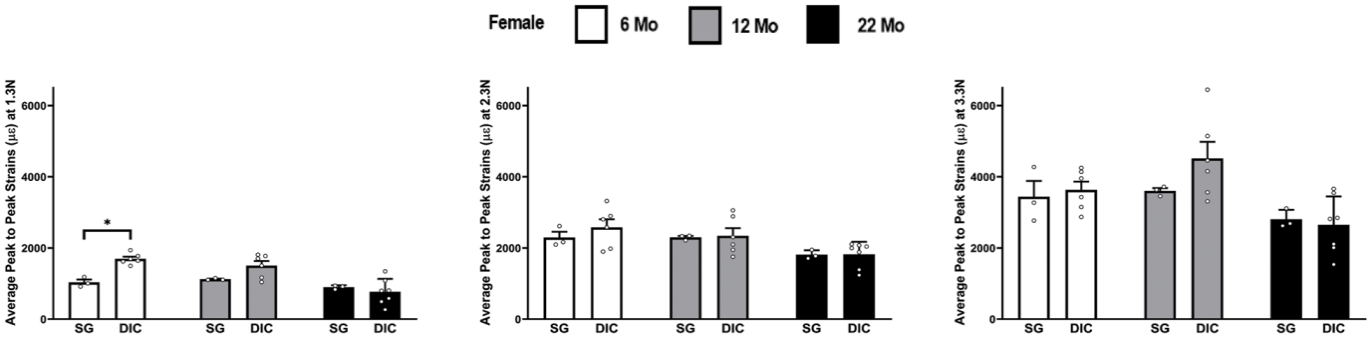
Strains captured by Digital Image Correlation (DIC) and Strain Gage in female ulnae (*p< 0.05) at load levels of 1.3 N, 2.3 N and 3.3 N.

The strains at three different load levels, of 1.3 N, 2.3 N and 3.3 N, using the DIC and strain gaging method in female ulnae are shown in Figure 4. DIC strains were higher compared to the strain gage values. However, the mean DIC strains were only found to be significantly higher in the 6 months age at a load of 1.3 N. The load strain relationship for female ulna is shown in Supplementary Figure 3.

### Male ulnae

**Figure 5:**
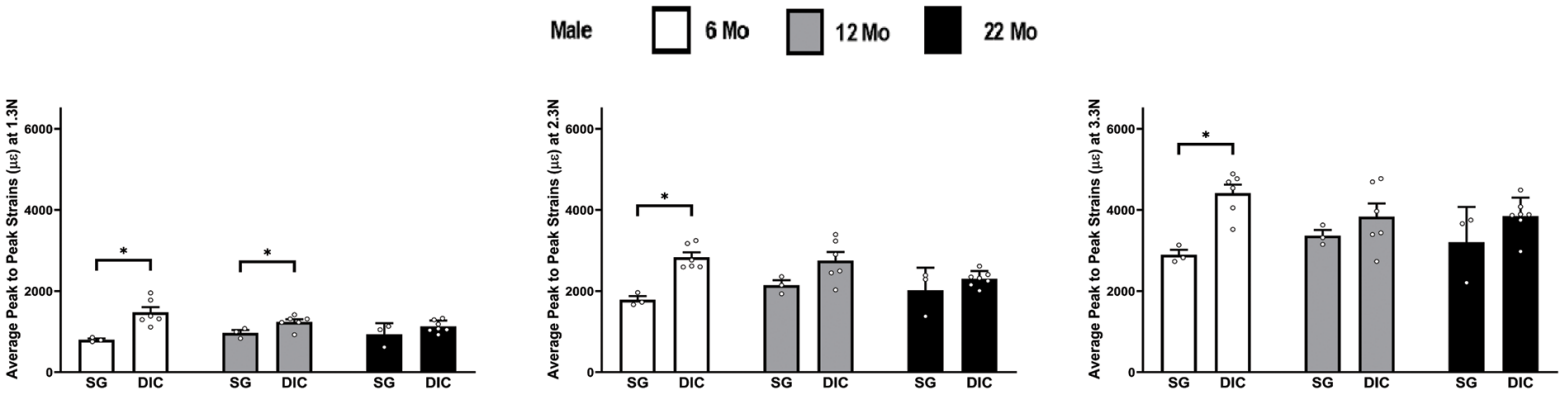
Strains captured by Digital Image Correlation (DIC) and Strain Gage in male ulnae (*p< 0.05) at load levels of 1.3 N, 2.3 N and 3.3 N.

The strains obtained from the DIC and strain-gaging method are shown in Figure 5, for male ulnae at three different levels of load. The DIC strains in male ulnae are significantly higher than strain gauge strains at 6 months at all load levels and at 12 months for 1.3 N only. The load strain relationship for male ulna is shown in Supplementary Figure 4.

### Effects of aging

### Tibia

**Figure 6:**
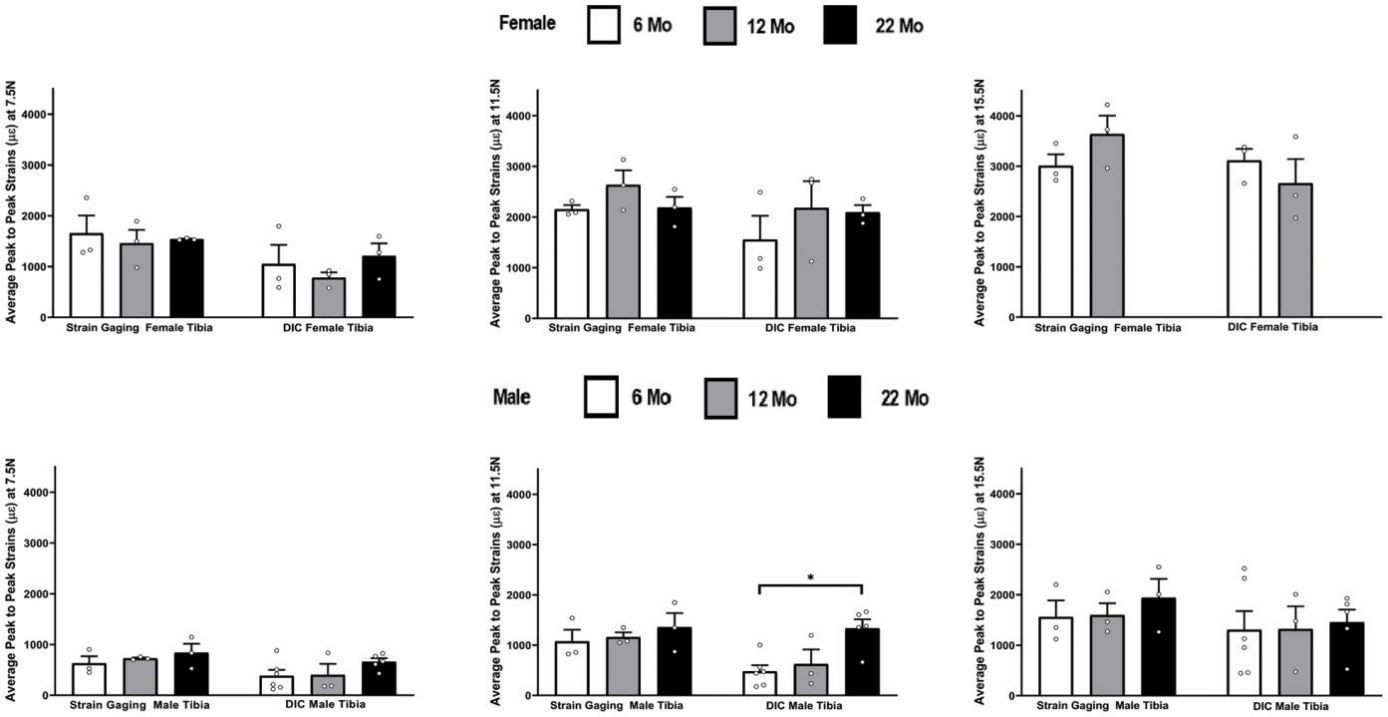
Average peak-to-peak strains captured from Strain Gage and DIC methods at 6 months, 12 months and 22 months groups tibiae (* p< 0.05).

The effects of aging was studied from the load-strain data and Figure 6 summarizes the strain differences in tibiae by age within each strain method, load level, and gender. The strain data for female in the 22 month old group was not included in Figure 6 because the samples started to break at the 15.5N load. The strain gaging method showed no significant differences in age within either female and male tibiae for any load level, while the DIC data only showed a significant increase in strains for male tibia from 6 months to 22 months at the load of 11.5 N.

**Figure 7:**
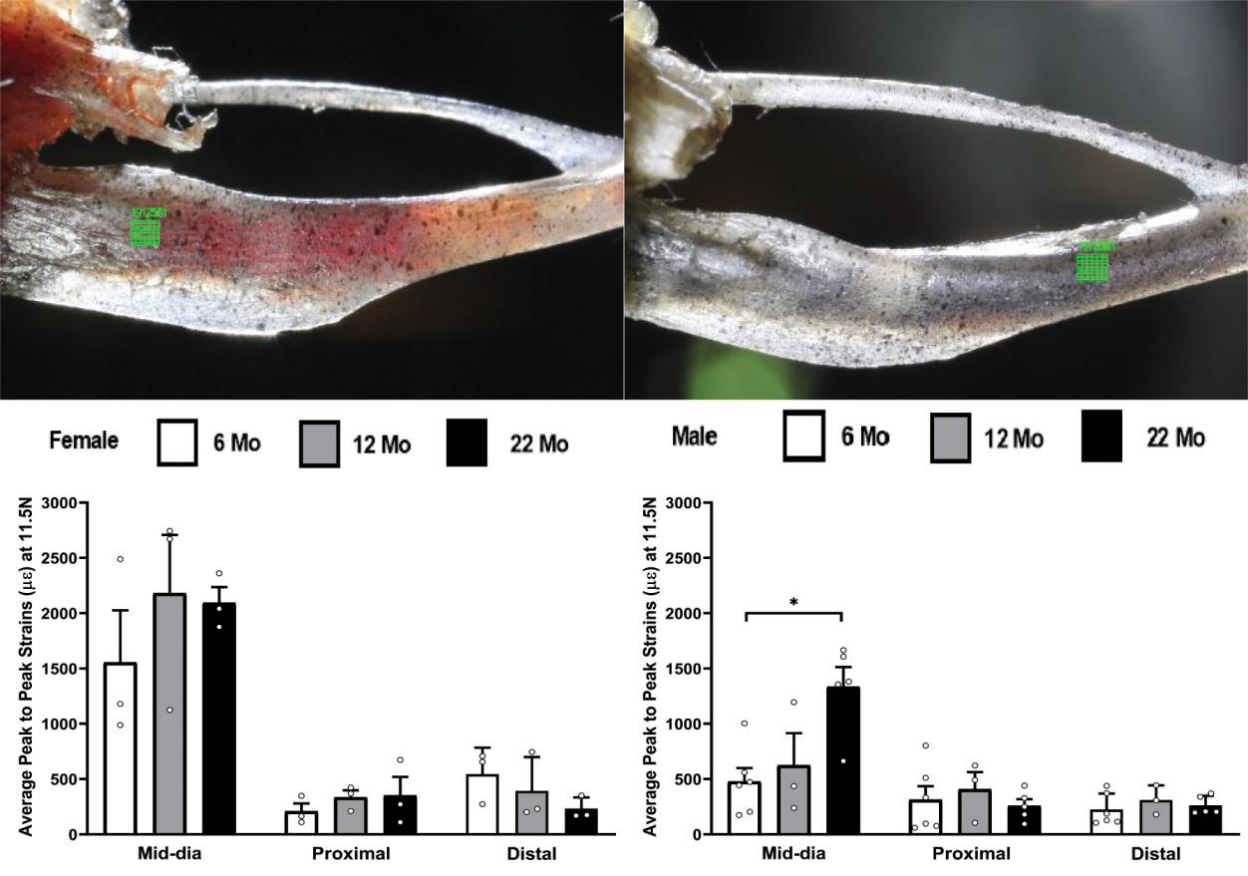
Average peak to peak strains captured from DIC method (n=3-6) at 6, 12 and 22 months groups for tibia at the mid-diaphysis shown in figure1(B), proximal and distal locations shown at the top left and top right respectively of the figure (* p< 0.05).

One of the advantages of the DIC method, compared to the strain gage method, is the capability of investigating changes in strain at different spatial locations using the same set of videos. We have also investigated the differences in strain behavior at different spatial locations for the tibia. Figure 7 shows the aveage peak-to-peak strain at 6, 12 and 22 month groups at the distal and proximal locations. Two observations from Figure 7 are that the strains at the proximal and distal locations are much lower than the mid-diaphysis region and no statistical differences with aging was observed at either of these locations. The decreased strains in these regions are primarily related to lower bending moment compared to the mid-diaphysis region.

### Ulna

**Figure 8:**
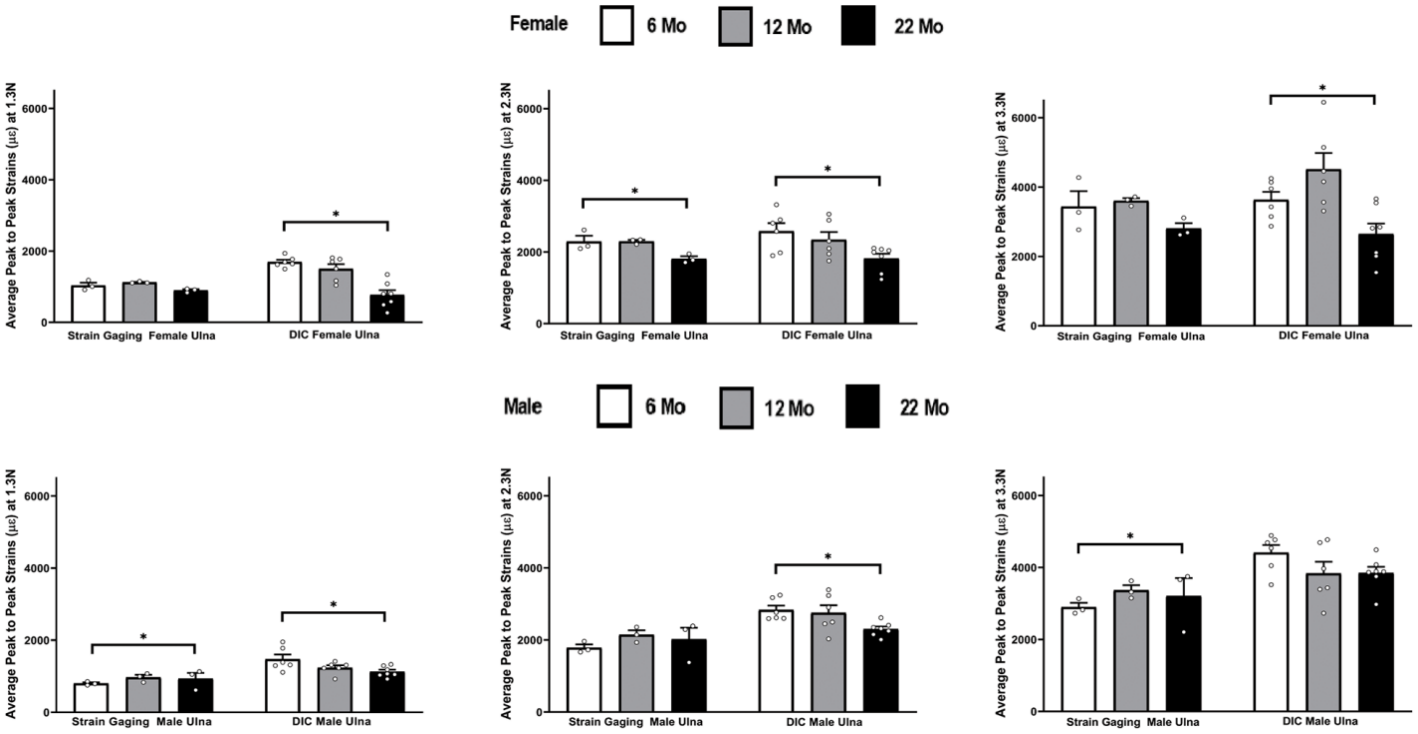
Average peak to peak strains captured from strain gaging (n=3) and DIC method (n=5-7) at 6, 12 and 22 months groups ulnae (* p< 0.05).

Figure 8 shows the strain differences in ulnae by age within each strain method, load level, and gender. The DIC strains decreased significantly from 6 to 22 months at all loads for females and at the two lowest loads for males. Strain gaging data showed significant increases in strains from 6 to 22 months at the lowest and highest loads for males and showed a significant decrease in strains from 6 to 22 months at the middle load level for females.

## DISCUSSION

The ulna forearm compression model and the tibia hind limb compression model have different strain distributions with respect to loading. Lee et al showed that ulna experiences compressive strains on the medial surface [18] and Sztefek et al demostrated that tibia experiences tensile strains on the medial surface due to its curved shape [15].

We observed that the strains captured by DIC technique in ulnae were significantly higher than the strain gage strains while tibia showed no significant differences in strains captured between the two methods. The morphology of the bone could also play an important role in the strains experienced on the surface of the bone. In addition, the single camera DIC technique works better with a flatter surface in general. Verhulp et al [19] and Franck et al [20] demonstrated that 2D-DIC method only captures a plane strain field corresponding to the sample surface. When the sample deforms out of plane or the sample is a non planar geometry, 3D-DIC technique would be a better approach. In the case of DIC, it is difficult to align the plane of the camera to the plane of the region of interest on the bone surface. Also, it is difficult to pick the region of interest from the surface aligned with the lens and also consistent with the location of the strain gage on the sample. Strain gaging has its own challenges also. Ramault et al determined that delamination can occur at the central portion of the bone during loading and this could effect the strain results [21]. Hence, care must be taken while attaching the strain gage on the bone surface. Since the ulna is morphologically more uniform and planar in its surface and compared to the tibia which has a non-planar geometry, it might explain why the ulna showed higher strains with DIC technique, but tibia did not show differences between the two methods.

In tibiae, there was no difference between the strains captured by both methods. A stiffer response was expected due to the application of the strain gage and glue, but was not observed. This could be in part due to the comparatively irregular surface of this bone or it could be due to the number of limitations associated with this technique. There are other factors that affect the determination of strain from the DIC method. Bornert et al determined that the DIC measurements are influenced by the values of a number of parameters required for the computation of strains [22]. Hensley et al showed that the quality of speckle pattern could directly impact the ability to capture accurate results [23].

The changes were evident in ulnae using either of the methods, while there are almost no changes observed in the tibiae with either DIC or strain gaging methods. Leucht et al showed that the strains on the medial surface of ulnar mid shaft increased as a function of load [18] and hence the medial surface of ulna can be considered as a best site to capture surface strains. Due to the flatter medial surface in ulnae compared to tibiae, DIC can be considered as a viable method to capture the bone surface strains in ulnae and similar flat bones.

The ulna is a bone that mostly consists of a cortical component, while the tibia represents a mixed model of cortical and trabecular bone. Chen et al concluded in human studies, that the trabecular bone loss is one of the most important age related bone changes [24]. Boskey et al has reviewed the age related changes in bone and found that trabecular number in bones decline significantly with aging in both humans and mice [1].

The tibiae showed no changes while ulnae showed a stiffer response with aging. Increase in stiffness of a bone is due to a combination of increased mineralization in the bone which affects the elastic modulus and increased architectural properties of the bone which is reflected in its cross section area and moment of inertia properties. We have recently shown that the cortical bone area in female tibia decreased significantly with aging while no differences were found for males [25]. No differences with aging in cortical bone area were found for either female or male ulnae. The elastic modulus for tibia showed no significant differences for both male and female mice while the elastic modulus of ulna increased significantly only for female ulnae from 6 to 22 months of age. The increase in elastic modulus for ulna could be one reason why the stiffness increased with aging for tibia but not ulna,

The presence of trabecular elements would favor structural flexibility over stiffness [26]. Since the tibia has both trabecular and cortical bone, it is likely that the increase in stiffness of the cortical compartment with aging could be offset by the changes in the trabecular properties. One study Heveran et al [27] studied the effect of chronic kidney disease (CKD) and aging on all male long bones (femur, tibia, ulna, humerus and radius for male mice aged 6,18 and 24 months). They reported that femurs showed a decrease in modulus and stiffness with aging. No data from either the ulna or tibia were reported. Another study by Main et al [26] conducted on 6, 10 and 16 week old female mice has also shown that the tibial stiffness decreased at 16 weeks of age in the loaded group of mice. This study may not represent an aging model at 16 weeks of age. Brodt et al [28] studied the mechanical properties of female femurs of mice upto the age of 24 weeks and reported that ultimate moment and bending rigidity increased up to 20 weeks and noted that material properties increased more than the cross-sectional geometric properties cross-sectional geometric properties.

Aging is the process of decline in biomechanical performance of the skeleton that occurs after reaching maturity. Overall, an imbalance in bone resorption and formation results in overall bone loss leading to increased fracture risk. However, a detailed aging study on the human skeleton is extremely difficult to accomplish and there is still insufficient understanding of how human bones age. Hence, an aging study of the mouse skeleton is presented in this study to ascertain changes in the performance of different bones.

This is a unique study performed on two different bones of the same mice. It provided an opportunity to perform direct comparisons of different bones of the same skeleton. The DIC technique had a higher sensitivity to detect statistical differences in strain response compared to strain gages. There are also important differences between the aging changes of the male mouse skeleton compared to the female mouse. A study on adaptive response to loading in mice tibia also revealed that the impairment to the bone with aging is different in males and female [28].

This study confirmed that predicted strains were higher using DIC compared to strain gaging for ulnae, but the tibiae displayed equivalent results by the two methods. However, more importantly the DIC method showed greater sensitivity compared to the strain gaging technique by detecting a greater number of parameters that were statistically significant.

## MATERIALS AND METHODS

### Equipment

The DIC system is composed of a loading machine (Bose Electroforce 3220, Bose Corp., ElectroForce Systems Group, Eden Prairie, MN) and two digital single lens reflex (DSLR) cameras. Both cameras (Canon Rebel T2i EOS 550D) were equipped with MP-E-65 mm macro photo lenses (Canon U.S.A. Inc. Melville, NY) which have a magnification ranging from 1x to 5x using manual focusing. Both cameras were mounted on to vertical adjustable columns bolted to an anti-vibration table. The columns are also equipped with sliding rails to adjust the distance of camera from the sample. LED light sources and external LCD monitors are also used for the optimization of the video quality. Loading caps specially designed for the particular type of the bones were installed in the Bose loading system [17].

### Ex vivo mechanical loading

For all the experiments, the Bose system applied a preload of 0.3 N to ulna specimens and 0.5 N load to tibia specimens. The ulnae were then dynamically loaded at 1.3 N, 2.3 N and 3.3 N at a frequency of 0.2 Hz for 5 cycles and the tibiae were loaded at 7.5 N, 11.5 N and 15.5 N at a frequency of 2 Hz for 40 cycles using a sinusoidal waveform.

High definition (HD) videos of loading were recorded at 30 frames per second. DIC strain values were calculated using a MATLAB-based specialized DIC software that tracks the speckle patterns from the videos of experiments and then computed the strains. The strain measured at different peak loads were compared across the gender and ages.

### Specimen preparation

C57Bl/6 mice were obtained from the NIH Mouse Aging Colony at Charles Rivers Laboratories. The mice were divided into three age groups: 6, 12 and 22 months. There were six males (weight 35.1 ± 2.4 g) and six females (weight 24.3 ±1.5 g) in the 6-month old group, six males (weight 37.4 ± 2.1 g) and six females (weight 27.9 ± 1.5 g) in the 12-month old group and seven males (weight 34.1 ± 3.8) and seven females (weight 30.3 ± 4.5) in the 22-month old group. The UMKC IACUC approved all animal studies.

The mice were euthanized by CO_2_ inhalation followed by cervical dislocation. Skin was removed from hind limbs and forearms and the soft tissues were removed from the left ulnae and right tibiae, and then wrapped in a PBS soaked gauze. The bones were stored in the -20° C freezer until needed.

Samples were thawed for 30-45 minutes before preparing them for the testing. Any residual soft tissue, muscle and ligaments were removed carefully from each of the sample using tweezers and scissors. Acetone was used to clean the surface of the bone to make the region of interest clearly visible. The samples were rehydrated with PBS and then speckled with a black, opaque, water-based paint (Createx Colors, East Granby, CT) using a high precision airbrush (Model 200NH Badger Air-brush Co., Franklin Park, IL) set to a pressure of 20 psi (138 kPa)*.* The paint was sprayed from a distance of approximately 6 inches. Three coats were applied to ensure a uniform speckle pattern over the lateral and medial surfaces of the bone. The samples were then observed under the microscope to see if the speckle pattern was clearly visible. This speckling procedure was similar to a procedure used in a previous study on mouse tibia to analyze the strain distribution [17].

### Digital image correlation (DIC)

The two-dimensional digital image correlation (2D-DIC) technique [17] was used and the images were analyzed with sub-pixel resolution. The 2D-DIC method typically measures the displacement vector acting on an image stack and then calculates the corresponding strains. A DIC code [29, 30] developed in MATLAB® (MathWorks, Inc., Natick, MA) was used to determine the strains in this study. Modification to the original code developed by Jones et al [29] was made by Begonia et al [17]. This code is used to determine the maximum strains for every sample based on the difference between the two adjacent peak values in a loading cycle and termed as peak-to-peak strains. Both DSLR cameras at 30 frames per second captured full HD videos (1980 x 1080-pixel resolution).

The cameras were placed in the medial and lateral viewing positions prior to loading. Each sample was secured between the loading caps while the macro photo lenses were set to the 1x magnification for ulnae and 2x magnification for tibiae. The samples were aligned with the axis of the loading caps and adjusted in order to ensure that the bone surface remained in focus. The videos of both medial and lateral sides were recorded simultaneously. The Bose loading system was activated with a delay of 30 seconds to allow the camera vibration caused by manual triggering to subside.

The videos were separated into individual frames using a custom MATLAB® script for further processing. The first loading frame, used as the reference frame, was determined by inspecting the movie and calculating the frame number using frames per second and time delay (30 sec x 30 frames/sec = 900 frames). Although the test protocol applied 5 cycles for ulnae and 40 cycles for tibiae, the peak-to-peak strain was averaged from the second, third and fourth cycles for ulnae from the nineteenth, twentieth and twenty first cycles for tibiae.

To compute the strains for the DIC experiments the region of interest (ROI) was selected as close as possible to the location where the strain gage was applied on the surface of the bone. This designation of ROI’s facilitated the direct comparison strain from DIC and strain gage measurement techniques. The ROI size was 128x96 pixels for ulnae and 85x64 pixels for tibiae. The size of the speckle was estimated about 4-6 pixels and the number of speckles were 50 to 100 per ROI.

### Strain gaging

Experimental strains were obtained from strain gages (EA-06-015DJ-120/LE Vishay Precision Group, Malvern, PA) attached to the medial ulnae and tibiae surfaces on the same samples that were used previously in the DIC experiments. The gage resistance was checked using a multimeter before and after the testing. Micro scissors and forceps were used to remove the muscle tissue and a cotton swab dipped in acetone was also used to remove any remaining soft tissue from the bone. This step was taken to ensure the proper attachment of the strain gage on the curved surface of the bone. The prepared wires were soldered with the wires coming from the StrainSmart Data Acquisition system (System 7000 Vishay Precision Group, Malvern, PA). The sample was placed in the loading fixture and a sinusoidal loading cycle was applied which was consistent to the loading applied for DIC method and the strains were recorded.

### Strain computation and statistical analysis

In Figures 2–8 the average strains for each age group were calculated at each load and compared between the two strain measurement techniques. The testing started with a preload of 0.5 N for the tibia and 0.3 N for the ulnae. The comparison of mean strain values between the two methods and between the age groups were made using linear regression models with robust standard errors. Significant differences (p<0.05) are marked with an asterisk (*) in the figures.

## Supplementary Material

Supplementary Figures
