# Supplementary material for: Age and gender related differences in load-strain response in C57Bl/6 mice
Source: Aging (Albany NY). 2020 Dec 17;12(24):24721–33. doi: 10.18632/aging.202350 (PMC7803533; doi:10.18632/aging.202350)
Supplement: Supplementary Figures [file aging-12-202350-s001.pdf]

SUPPLEMENTARY FIGURES

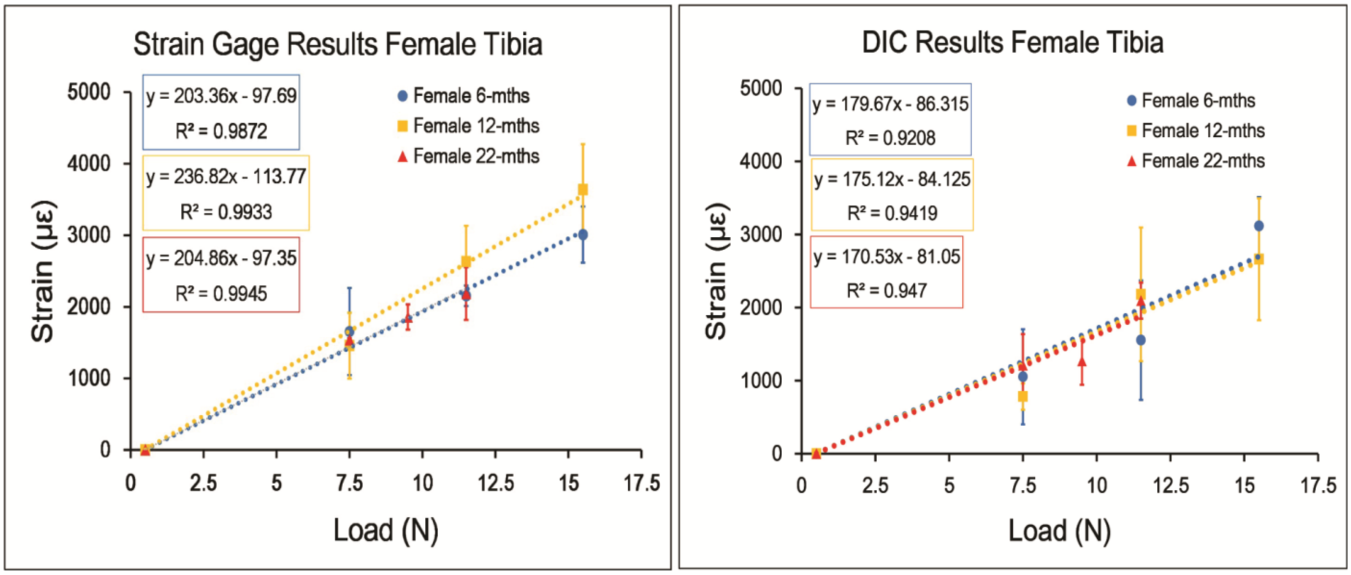

Supplementary Figure 1. Strains captured by Strain Gage and Digital Image Correlation (DIC) in female tibiae (data are mean ± standard error).

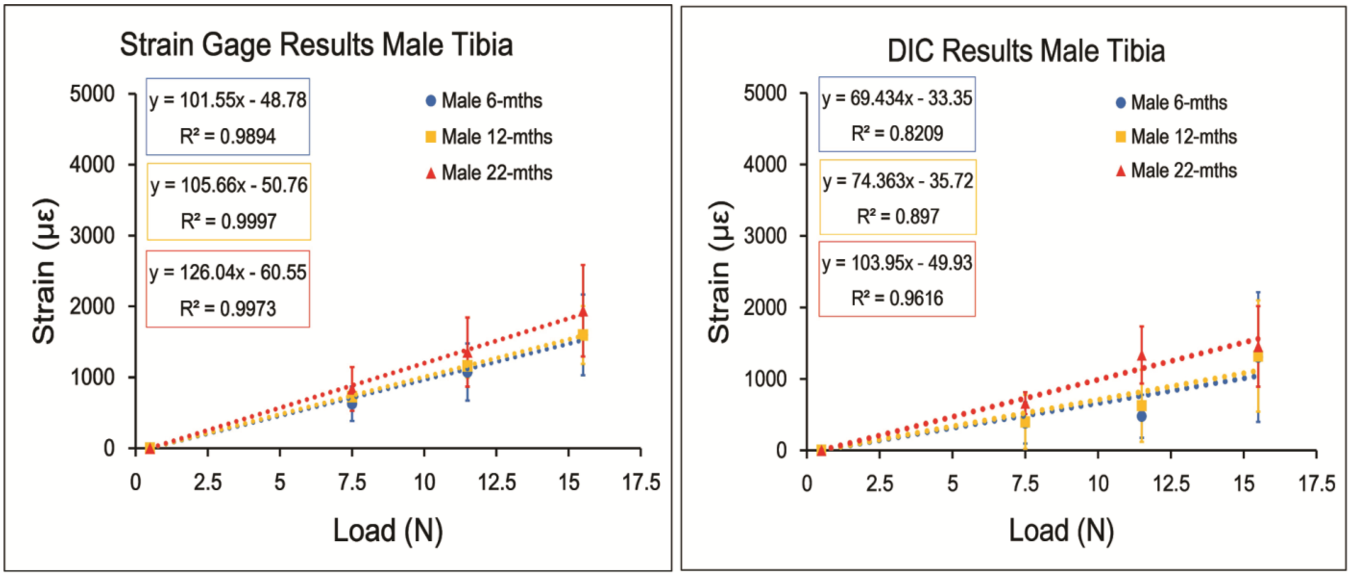

Supplementary Figure 2. Strains captured by Strain Gage and Digital Image Correlation (DIC) in male tibiae.

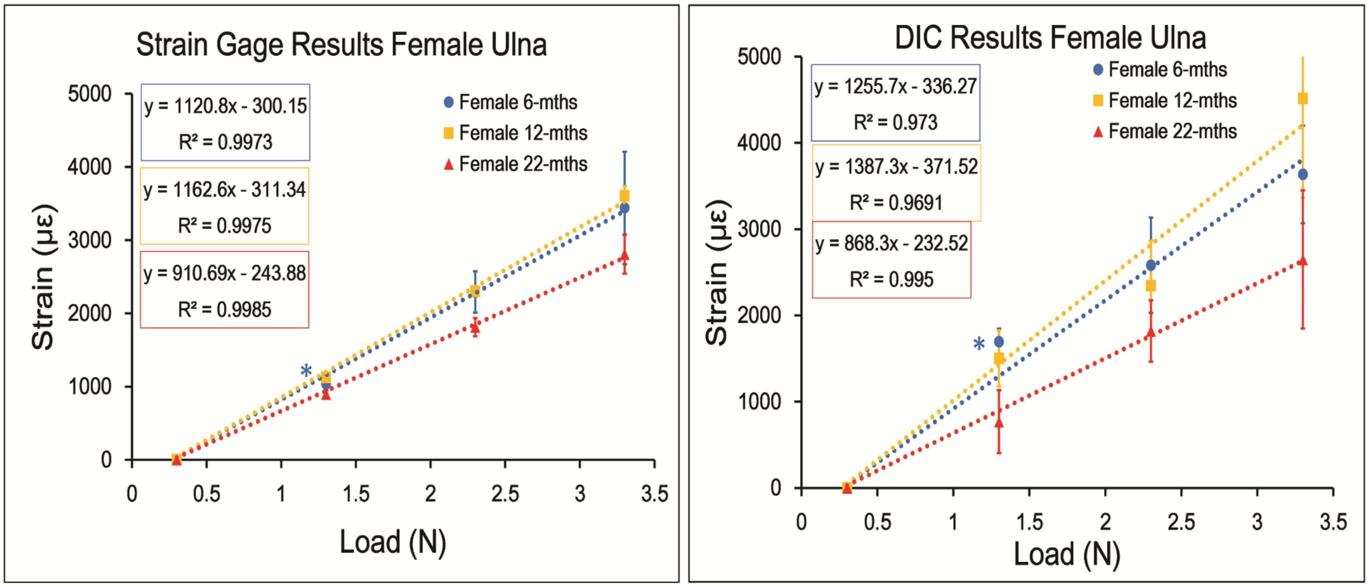

Supplementary Figure 3. Strains captured by Strain Gage and Digital Image Correlation (DIC) in female ulnae.

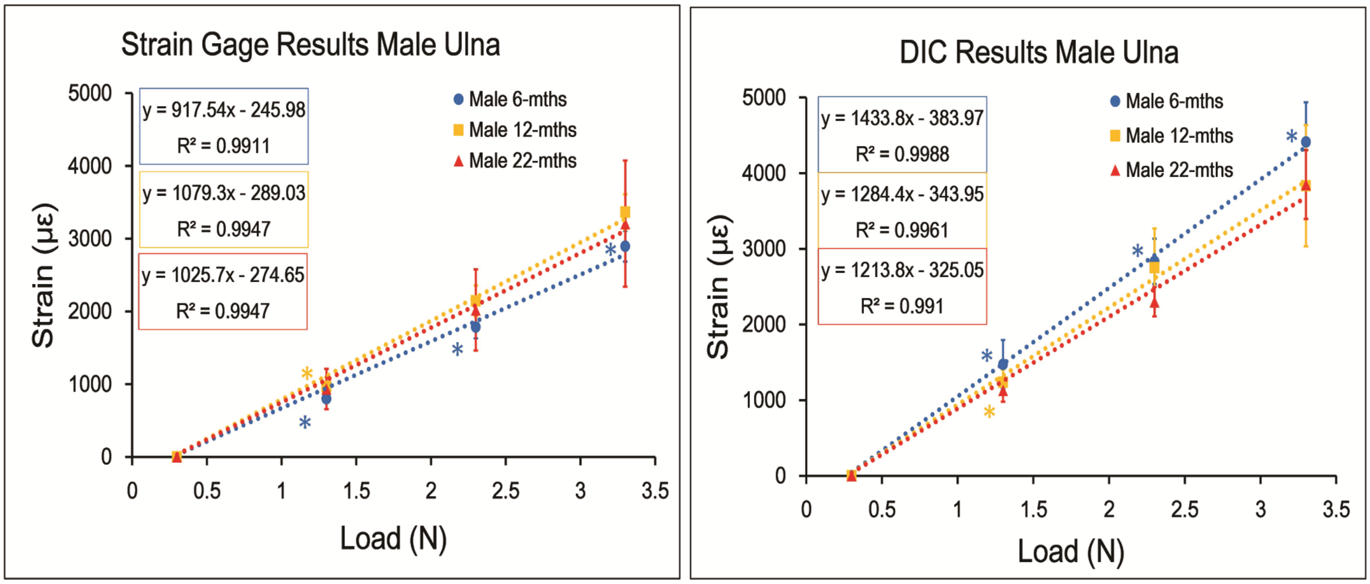

Supplementary Figure 4. Strains captured by Strain Gage and Digital Image Correlation (DIC) in male ulnae.
